# A challenging case of ALK-negative anaplastic large cell lymphoma in a 12-year-old boy: A rare case report from Syria

**DOI:** 10.1016/j.amsu.2022.104085

**Published:** 2022-06-25

**Authors:** Seif-Aldin Abdul Rahman, Karam Loutfi, Tareq Turk, Ali Abdul Rahman, Haidara Kherbek, Abdulmoniem Ghanem, Zuheir Alshehabi

**Affiliations:** a Faculty of Medicine, Cancer Research Center, Tishreen University, Latakia, Syria; b Department of Pathology, Tishreen University, Latakia, Syria; c Department of Pediatrics, Faculty of Medicine, Tishreen University, Latakia, Syria; d Department of Pathology, Cancer Research Center, Tishreen University, Latakia, Syria

**Keywords:** Anaplastic large-cell lymphoma, Non-hodgkin lymphoma, CD30, Pediatric, Case report

## Abstract

**Introduction and importance:**

Anaplastic Large-cell Lymphoma (ALCL) is a rare but aggressive type of NHL that develop from mature post-thymic T-cells. ALCL constitutes approximately 2% of all lymphoid neoplasm. It is typically found among children and young adults, accounting for 10–15% of pediatric NHL, compared to 2% of adult NHL.

**Case presentation:**

A 12-year-old Syrian boy was admitted to our hospital due to epistaxis, anorexia, weight loss and night sweats. The physical examination revealed preauricular, postauricular and submandibular lymphadenopathy. Pathological examination of the biopsy suggested Classical Hodgkin Lymphoma. Later on, Immunohistochemistry staining confirmed the diagnosis of ALK-negative Anaplastic Large Cell Lymphoma.

**Clinical discussion:**

Systemic ALCL can be categorized into two major groups based on the expression of Anaplastic Lymphoma Kinase (ALK) protein: Systemic ALK + positive and Systemic ALK-negative. The majority of pediatric cases show an overexpression of (ALK), however, pediatric ALK-negative ALCL can occur in rare cases.

**Conclusion:**

The aim of this article is to report a rare case of pediatric ALK-negative anaplastic large cell lymphoma that developed a rapid & aggressive growth within a few months despite the chemotherapy treatment and unfortunately led to the patient's death.

## Highlights

• Anaplastic large cell lymphoma (ALCL) is a rare type of non-Hodgkin lymphoma and one of the subtypes of T cell lymphoma.
• ALK-negative ALCL is rare among children and has a poor prognosis.
• Establishing ALCL diagnosis is challenging due to the similarities with CHL, DLBCL and PTCL-NOS.

## 1 Introduction

Anaplastic Large-cell Lymphoma (ALCL) represents a rare group of T-cell Lymphoproliferations that share morphological and immunophenotypical features, specifically strong CD30 expression and variable loss of T-cell markers. It was first described by Stein et al., in 1985 [1].

According to the 2016 revised world health organization (WHO) classification, ALCL is classified into four entities: Systemic ALK-positive, Systemic ALK-negative, Primary cutaneous ALCL and Breast implant-associated ALCL [2].

ALK is rearranged in approximately 80% of systemic ALCL, which is more common among children and has a relatively good prognosis, whereas ALK-negative ALCL occurs predominantly in adults (40–65 years) and has a worse prognosis [2,3].

Therefore, we present a rare case of pediatric ALK-negative ALCL that was initially misdiagnosed as a Classical Hodgkin Lymphoma.

## 2 Case presentation

**Fig. 1:**
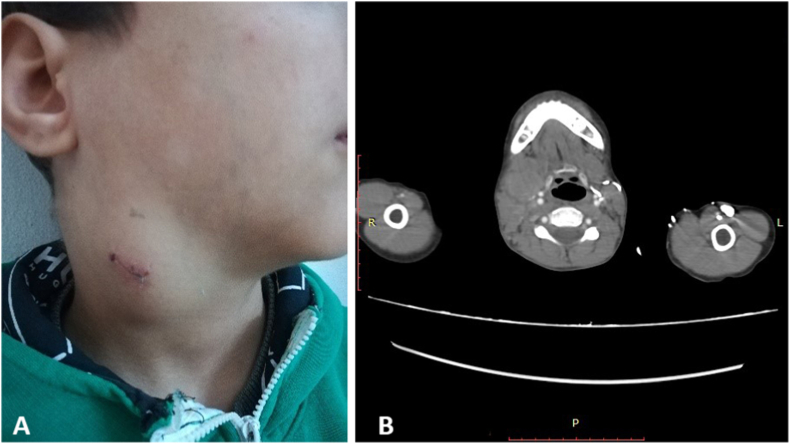
**(A)**: Clinical view of the patient showing right lateral cervical mass measuring approximately (3 × 5) cm. **(B):** CT scan in axial section revealing enlarged lymph nodes measuring approximately (44x38)mm on the right side of the neck.

A 12-year-old Syrian boy was admitted to our hospital due to epistaxis, anorexia, weight loss, and night sweats. Medical and family history were unremarkable. The physical examination revealed preauricular, postauricular and submandibular lymphadenopathy with the largest node measuring approximately (3 × 5) cm (Fig. 1). Furthermore, a hard palate mass was found deviating the uvula to the right causing dysphagia and dyspnea. Laboratory tests showed WBC count 35380/μL, Hgb 10,1 g/dl, platelets 641 × 10^3^/μL and ESR 124 mm/hr.

Abdominal ultrasound showed mild spleen enlargement without focal lesions. Chest radiography was normal without mediastinal widening.

A CT scan revealed multiple enlarged lymph nodes on the right side of the neck (Fig. 1).

**Fig. 2:**
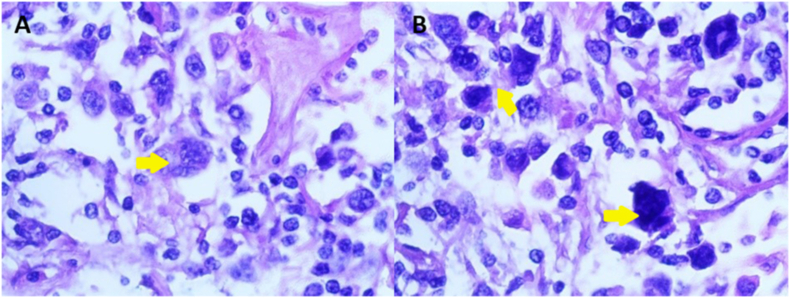
**(A):** sections of resected lymph node exhibiting complete effacement of lymph node architecture with Proliferation medium to large-sized anaplastic and Reed-Sternberg like cells(arrow) admixed with histiocytes & small lymphocytes(H&E, original magnification x400) **(B):** The neoplastic cells showing a cohesive growth pattern with abundant cytoplasm, multiple nuclei, multiple nucleoli, and occasional mitotic figures. (H&E original magnification x400).

Excisional biopsy of the enlarged cervical lymph node (3 × 4)cm was performed and further pathological assessment showed complete effacement of lymph node architecture with proliferation medium to large-sized anaplastic and Reed-Sternberg-like cells in the interfollicular zones and subcapsular sinuses admixed with histiocytes, small lymphocytes and few eosinophils. In addition, the neoplastic cells showed cohesive growth pattern with abundant cytoplasm, wreath-like or multiple nuclei, multiple nucleoli, and occasional mitotic figures (Fig. 2).

In addition, a bone marrow sample showed reactive inflammation with hypercellular bone marrow. No evidence of involvement by neoplastic cells was found. Consequently, the patient was diagnosed with Classical Hodgkin Lymphoma (CHL) based on routine staining with the recommendation to proceed with immunohistochemistry staining to confirm the diagnosis. However, immunohistochemistry was not available in our institution at the time.

**Fig. 3:**
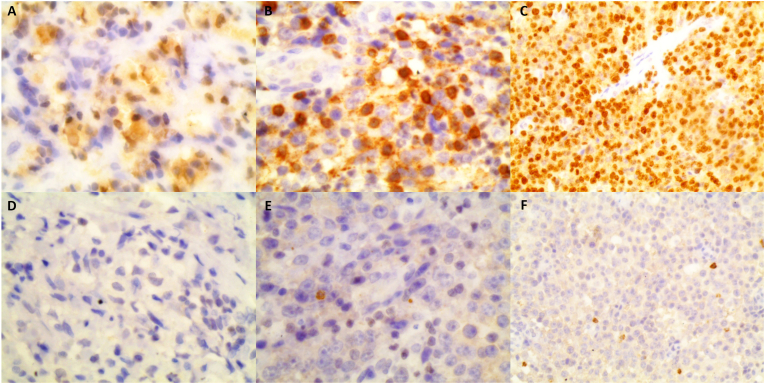
Immunohistochemistry of the lymph node: **(A):** strong CD30 positivity for neoplastic large and Reed–Sternberg-like cells; **(B):** The neoplastic cells are positive for CD3; **(C):** The neoplastic cells showing a high proliferation rate (Ki67 ≈ 70–80%); **(D):** The neoplastic cells are negative for ALK; **(E):** The neoplastic cells are negative for CD20; **(F):** The neoplastic cells are negative for CD15.

The patient received chemotherapy based on ABVD protocol. However, the patient reentered our hospital 5 months later with no evidence of significant improvement, therefore, the diagnosis was doubted. Consequent immunohistochemistry staining showed strong CD30 positivity for large Reed-Stenberg-like cells and CD3 positivity whereas ALK, CD15 and CD20 were negative (Fig. 3). Based on these findings, the patient was diagnosed with ALK-negative ALCL. The patient received 5 cycles of chemotherapy comprising of (vinblastine 10 mg/m^2^, methotrexate 500 mg/m^2^, ifosfamide 1g/m^2^, etoposide 100 mg/m^2^and cytarabine 1g/m^2^). Unfortunately, the patient passed away after 6 months.

## 3 Discussion

Anaplastic large cell lymphoma (ALCL) is a rare type of non-Hodgkin lymphoma (NHL), and one of the subtypes of T cell lymphoma. ALCL comprises about one percent of all NHLs and approximately 16% of all T cell lymphomas. ALCL is mainly divided into cutaneous and systemic types. Based on the expression of ALK protein, Systemic ALCL was divided into two distinct entities: ALK + ALCL, and ALK- ALCL [4,5]. ALK-negative ALCL typically affects adults with comparatively poor prognosis, whereas our case was diagnosed in a 9-year-old boy [6]. Patients diagnosed with ALK + ALCL were found to be of higher survival rates. This may be due to the cytotoxic T lymphocytes, helper T cells and B cells response in the patient immune system [7].

Systemic ALCL affects mostly lymph nodes with frequent extranodal sites including skin, bone, soft tissue, lung, liver and spleen. The involvement of the gastrointestinal tract or central nervous system is rare [8,9].

Morphologically, ALCL revealed heterogeneity showing a large and pleomorphic, small-cell, Hodgkin-like or Sarcomatoid appearance [3]. There are currently five morphological patterns that are described based on the variability of cell size, its morphology and its histological characteristics. All morphological patterns share the presence of large lymphoid cells with abundant cytoplasm and pleomorphic, often horseshoe - or kidney-shaped nuclei, which have been called “Hallmark cells” [3]. Frequently, Multinucleated large cells can be present and have large, more eosinophilic nuclei similar to Reed-Sternberg cells, as in our case, creating a diagnostic confusion with CHL [1].

Establishing ALCL diagnosis is challenging due to the similarities with CHL, DLBCL and PTCL-NOS, which include the proliferation of large pleomorphic cells with abundant cytoplasm and large atypical nuclei. However, CHL has mixed inflammatory background with classic large Reed-Sternberg cells. Moreover, DLBCL can be recognized by the B-cells lineage, whereas PTCL-NOS is distinguished with its small clusters of epithelioid histiocytes [5,10].

Immunohistochemistry staining in ALCL shows an expression of CD30 which is usually noticed at the cell membrane and in the Golgi region. However, CD30 is not specific for ALCL [2]. In addition, CD45, CD45ro. CD2, CD4 and CD5 are variably positive in ALCL demonstrating T-cell lineage [8,9]. Interestingly, the tumor cells have unusual proteins like cytotoxic TIA+, granzyme B+, perforin+, granulysin+ and EMA + which presumed the cytotoxic T-cell origin of ALCL. However, in some cases, no specific T-cell proteins can be detected and these are categorized as “null cells”, which is similar to our case [1]. Furthermore, the negativity for CD 15 excluded the diagnosis of CHL in our case whereas the negativity for CD20 excluded DLBCL. Also, PTCL-NOS was ruled out due to the negativity of both CD3 and CD15.

Molecularly, the classic t(2; 5) (p23; q35) generates a fusion between nucleophosmin gene(NPM) and ALK. This is a common hallmark of ALCL and is usually seen in 40–60% of cases [9]. Other translocations include t(1; 2) (q25; p23) inv(2) (p23; q35), t(2; 3) and a CLTCL-ALK fusion transcript typically resulting from a t(2; 17). ALK + ALCL also includes overexpression of HIF1-a target genes with H-ras/K-ras induced genes, whereas ALK- ALCL shows recurrent chromosomal gains in 46% of cases, with losses of 6q and 13q both occurring in 23% of cases [9]. Moreover, DUSP22 rearrangements, activating JAK1/STAT3 mutations and rearrangements leading to TP63 mutations have also been reported in 30%, 20%, and 8% of cases, respectively [2,10]. Unfortunately, molecular tests are not available in our hospital, therefore, the diagnosis was confirmed based on IHC staining and morphological finding.

## 4 Conclusion

Our article demonstrates a challenging case of ALK-negative ALCL, characterized by its aggressive and rapid progression; which lead to a limited response to chemotherapy and subsequently a very poor prognosis. Our article also highlights the important role of histopathological and immunohistochemical examinations in the diagnosis & differential diagnosis of this unique entity.

## Ethical approval

Not applicable.

## Sources of funding

None.

## 5 Contributions

SA: drafted the manuscript KL and TT: participated in drafting the manuscript and collected the patient's data. AA: participated in drafting the manuscript and revised the manuscript. HK: participated in revising the manuscript.AG: The pediatric oncologist: was in charge of the patient's treatment and status.ZA: The mentor and guarantor, performed the pathological examination, critically revised the article and approved the final manuscript.

## 6 Registration of research studies

Not applicable.

## Guarantor

Dr. Zuheir Alshehabi.

## Consent

Written informed consent was obtained from the patient's parents for publication of this case report and accompanying images. A copy of the written consent is available for review by the Editor-in-Chief of this journal on request.

## Availability of data and materials

Data and material are available on reasonable request from the guarantor and mentor of this study Prof. Zuheir Alshehabi.

## 7 Provenance and peer review

Not commissioned, externally peer-reviewed.

## Declaration of competing interest

None.

## Ethical approval

Not applicable. It's a case report.

## Sources of funding for your research

None declared. The research didn't receive any funding.

## Author contribution

SA: drafted the manuscript KL and TT: participated in drafting the manuscript and collected the patient's data. AA: participated in drafting the manuscript and revised the manuscript. HK: participated in revising the manuscript.AG: The pediatric oncologist: was in charge of the patient's treatment and status.ZA: The mentor and guarantor, performed the pathological examination, critically revised the article and approved the final manuscript.

## Consent

Written informed consent was obtained from the patient's father for publication of this case report and accompanying images. A copy of the written consent is available for review by the Editor-in-Chief of this journal on request.

## Registration of research studies

Not applicable. It's a case report.

## Guarantor

Dr. Zuheir Alshehabi.

## Declaration of competing interest

None declared.
